# Large Virtual Transboundary Hazardous Waste Flows: The Case of China

**DOI:** 10.1021/acs.est.2c07962

**Published:** 2023-05-16

**Authors:** Ruoqi Li, Miaomiao Liu, Yuli Shan, Yufan Shi, Heran Zheng, Wei Zhang, Jianxun Yang, Wen Fang, Zongwei Ma, Jinnan Wang, Jun Bi, Klaus Hubacek

**Affiliations:** † State Key Laboratory of Pollution Control and Resource Reuse, School of the Environment, Nanjing University, Nanjing 210023, People’s Republic of China; ‡ School of Geography, Earth and Environmental Sciences, University of Birmingham, Birmingham B15 2TT, U.K.; § The Bartlett School of Sustainable Construction, University College London, London WC1E 7HB, U.K.; ∥ State Environmental Protection Key Laboratory of Environmental Planning and Policy Simulation, Chinese Academy of Environmental Planning, Beijing 100041, People’s Republic of China; ⊥ Integrated Research on Energy, Environment and Society (IREES), Energy and Sustainability Research Institute Groningen (ESRIG), University of Groningen, Groningen 9747 AG, The Netherlands

**Keywords:** hazardous waste, Basel Convention, input−output analysis, trade, virtual flows, sustainable consumption, China

## Abstract

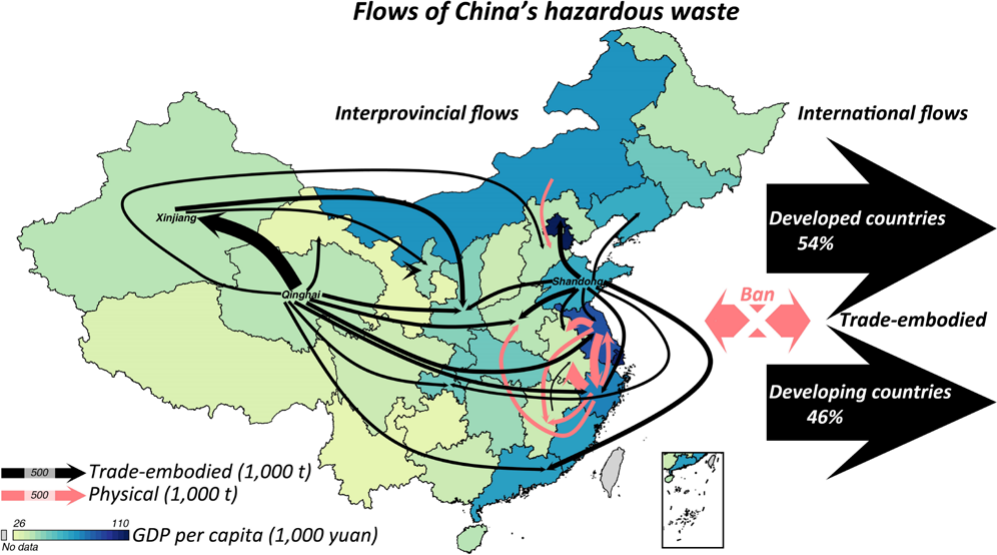

The Basel Convention and prior studies mainly focused on the physical transboundary movements of hazardous waste (transporting waste from one region to another for cheaper disposal). Here, we take China, the world’s largest waste producer, as an example and reveal the virtual hazardous waste flows in trade (outsourcing waste by importing waste-intensive products) by developing a multiregional input–output model. Our model characterizes the impact of international trade between China and 140 economies and China’s interprovincial trade on hazardous waste generated by 161,599 Chinese enterprises. We find that, in 2015, virtual hazardous waste flows in China’s trade reached 26.6 million tons (67% of the national total), of which 31% were generated during the production of goods that were ultimately consumed abroad. Trade-related production is much dirtier than locally consumed production, generating 26% more hazardous waste per unit of GDP. Under the impact of virtual flows, 40% of the waste-intensive production and relevant disposal duty is unequally concentrated in three Chinese provinces (including two least-developed ones, Qinghai and Xinjiang). Our findings imply the importance of expanding the scope of transboundary waste regulations and provide a quantitative basis for introducing consumer responsibilities. This may help relieve waste management burdens in less-developed “waste havens”.

## Abstract 2

Research mainly focuses on direct transboundary movements of hazardous waste between regions. This study reveals virtual hazardous waste flows that can exceed physical transboundary waste movement.

## 1 Introduction

Waste management is one of the most daunting challenges facing the world today.^1,2^ Over half of the United Nations Sustainable Development Goals (SDGs) are related to waste management issues.^3,4^ Hazardous waste (i.e., waste with toxicity, corrosivity, ignitability, reactivity, or infectivity) may significantly harm the environment and human health, and thus should be prioritized in waste management.^5,6^ 300–500 million tons (mt) of hazardous waste are produced globally every year.^7^ Awakening environmental awareness and ensuing tightening of environmental regulation in developed regions end up increasing public resistance to disposal facilities (i.e., Not In My Back Yard). Outsourcing hazardous waste disposal services and exporting used appliances containing hazardous components (i.e., physical waste transfers) to areas with cheap disposal options and lax regulations have provided an outlet for hazardous waste management in developed regions.^8−10^ As a result, waste burdens are often disproportionately suffered by poorly regulated developing economies. To address such inequalities, the United Nations adopted *the Basel Convention on the Control of Transboundary Movements of Hazardous Wastes and their Disposal* (herein referred to as “*the Basel Convention*”),^11^ restricting hazardous waste movements across borders, especially those from developed countries to developing ones.

China plays a significant role in addressing global hazardous waste issues. China’s economic miracle made it the world’s largest waste producer. The annual growth rate of hazardous waste generation in China is even 4 percent higher than that of its economy (measured by gross domestic product, GDP).^12,13^ Meanwhile, China was the leading importer of global waste as well.^14,15^ A decade ago, up to 70% of the world’s e-waste ended up in China,^16^ exacerbating challenges of hazardous waste management in China. In this context, in 2017, the Chinese government enacted an unprecedented ban (i.e., *Prohibiting the Imports of Foreign Garbage: the Reform Plan on Solid Waste Import Management*, herein referred to as “*the Chinese import ban*”), restricting the import of many types of waste from other countries.^14,17,18^ Furthermore, in response to interprovincial physical transfers, China has implemented stricter policies to monitor the cradle-to-grave process of hazardous waste.^19^ Under the policy, hazardous waste producers are expected to minimize waste at the source.^20,21^ However, expanding and increasingly complex trade networks offer another option. That is, developed regions may comply with transboundary waste regulations by avoiding transporting their local hazardous waste to other regions; instead, they may directly import waste-intensive products and raw materials from less-developed areas. Such interregional trade does not only impact waste disposal processes but also transfers activities generating hazardous waste from the consuming region to the producing region. Since the disposal duty of hazardous waste belongs to the generator, the virtual transfer through trade implies a more radical transfer of waste management responsibilities than that caused by the physical waste transfer. In this context, it is necessary to shift the focus of waste management from end-of-life disposal to the entire supply chain.^4^

Existing studies have investigated the virtual hazardous waste network among industries and final demand categories within single economies, including the US,^22−24^ Spain,^25,26^ Germany,^27^ Belgium,^28^ France,^29^ Australia,^30^ the UK,^31^ South Korea,^32^ and several individual provinces in China.^33,34^ Yet less is known about the virtual hazardous waste transfers between regions at different stages of development.^35^ Given the increasingly widening geospatial separation of producers and consumers,^36,37^ reduced hazardous waste in some developed regions is likely achieved by outsourcing production activities and associated waste to less-developed regions. Since dumping industrial hazardous waste is illegal, hazardous waste accumulation can be particularly pronounced in less-developed regions lacking advanced technologies and sufficient disposal capacities. Therefore, developing hazardous waste management strategies with the consideration of trade-embodied virtual transfers between different regions may provide a potential path toward hazardous waste minimization in these hotspot regions.^38,39^

Here, we uncover the substantial virtual flows of hazardous waste in China’s trade beyond the transboundary physical movements, by developing an environmentally extended multiregional input–output (EEMRIO) model. Our model incorporates hazardous waste generated by 161,599 Chinese enterprises in 573 sectors and characterizes the transfers of hazardous waste embedded in international trade between China and 140 other economies as well as interprovincial trade between 31 Chinese provinces in 2015. Our results and research framework provide a quantitative basis for implementing consumer responsibilities in various transboundary waste regulations, thereby helping to encounter waste challenges in hotspot regions in China and other emerging economies that may be considered the next “waste havens”.

## 2 Materials and Methods

Based on China’s most detailed firm-level environmental database, covering all types of hazardous waste defined by the Chinese government,^40^ from 161,599 enterprises in 573 sectors based on the 4-digit National Standard Industrial Classification (NSIC), we first compile China’s industrial hazardous waste inventory, presenting waste information for 31 provinces and 26 aggregated industrial sectors in 2015 (see Table S1 in the Supporting Materials). Then, we establish an EEMRIO model by incorporating the inventory into a globally integrated multiregional input–output (MRIO) model.^41^ The consumption- and production-based hazardous waste generation in each province and hazardous waste embodied in China’s international and domestic trade are estimated under the MRIO framework.

### 2.1 China’s Hazardous Waste Inventory

China is playing a crucial role in global efforts to tackle hazardous waste, but uncertainties in China’s hazardous waste generation inventory remain high. Existing studies mainly focused on waste generation at the national scale.^42,43^ The limited attempts at the provincial level mostly used the national situation as a proxy to disaggregate a province’s total hazardous waste generation into different sectors.^34,44^ Therefore, developing a hazardous waste inventory with high spatial resolution and sectoral coverage is the first step in investigating the virtual waste transfer in trade.

We obtained the data of 161,599 enterprises in 573 NSIC industries from the 2015 China’s Environmental Statistics Database (CESD). The CESD is the most authoritative and reliable nationwide environmental survey database, incorporating all major industrial pollution sources in China. All information in the CESD is self-reported by enterprises following the guidelines from China’s Ministry of Ecology and Environment (MEE). The local ecology and environment bureaus ensure the authenticity of the data through regular monitoring and unannounced on-site surveys and submit the corrected version to provincial departments for review. A series of regulations and laws stipulate the above-mentioned data quality control procedures.

The CESD is particularly strong in characterizing China’s industrial hazardous waste, since the MEE mandates reporting by all industrial enterprises that generate waste with hazardous characteristics (including more than 450 types of hazardous waste recorded by the Chinese government).^40^

As preparation for subsequent integration with the MRIO model, we aggregate the processed firm-level data into a hazardous waste inventory that distinguishes 31 provinces and 26 industrial sectors in China. The bridging between the 4-digit NSIC industries and the 26 industrial MRIO aggregated sectors is shown in Table S2 in the Supporting Materials. Table S3 demonstrates the hazardous waste inventory.

### 2.2 Environmentally Extended Multiregional Input–Output Analysis

The EEMRIO model is a powerful tool for exploring the virtual transfer of various environmental consequences (e.g., carbon emissions,^36,45,46^ air pollution,^47−49^ water consumption,^50−52^ and land use^53,54^) through trade networks. However, the impact of interregional trade on waste generation, especially hazardous waste, is far less discussed. Nakamura and Kondo^55^ expanded the standard environmental input–output model to the waste input–output (WIO) model, by relaxing the strict one-to-one correspondence between waste types and treatment technologies. However, the Chinese data on hazardous waste is not yet specific enough to build a WIO differentiating situations in each province. Meanwhile, the WIO more specifically describes the waste treatment and recycling processes at the end-of-life stage of products and is thus particularly strong in depicting a circular system comprising multiple industries in an economy;^56−58^ while our research focuses on the virtual flows of hazardous waste among different regions. Therefore, in this study, we construct an EEMRIO model, treating the hazardous waste generation in our inventory as a satellite account. Specifically, we integrate a row of numbers representing hazardous waste generated during production activities in different economic sectors (i.e., the satellite account) into a conventional MRIO table.^59^

We obtained the basic globally integrated MRIO model from Zheng et al.,^41^ which characterizes trade between 42 sectors in 31 Chinese provinces and 57 sectors in 140 other economies by connecting China’s MRIO table with the GTAP-MRIO model (see details in Table S4 in the Supporting Materials). Compared with other global nested models, the model we employ preserves the authenticity of China’s interprovincial trade to the greatest extent. When linking the two MRIO tables, Zheng et al. keep China’s MRIO unchanged and adjust the GTAP-MRIO model based on the RAS approach (a data reconciliation approach that bi-proportionally scales a matrix to satisfy pre-specified row and column sum constraints). Since our research mainly focuses on China, this model is more applicable than other global nested models.

The framework of the nested GTAP-MRIO model can be expressed as follows
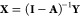
1
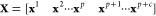
2
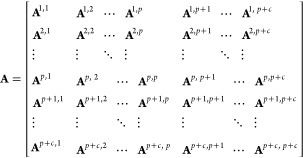
3
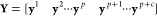
4where matrix **X** is the total output matrix; its element **x**^*r*^ = {*x*_*k*_^*r*^} is a column vector representing the total output of the individual sector *k* (i.e., each sector of each region) induced by the final demand in region *r*; matrix **I** denotes an identity matrix; **A** refers to the technical matrix; its element **A**^*r,s*^ = {*a*_*i,j*_^*r,s*^} stands for the technical submatrix depicting the relationship between region *r*’s intermediate supply and region *s*’s intermediate demand, given by *a*_*i,j*_^*r,s*^ = *z*_*i,j*_^*r,s*^/*x*_*j*_^*s*^, in which *z*_*i,j*_^*r,s*^ represents the monetary flow from sector *i* in region *r* to sector *j* in region *s*; **Y** represents the final demand matrix; its element **y**^*r*^ = {*y*_*k*_^*r*^} is a column vector indicating the supply of individual sector *k* for final consumption in region *r*; *p* = 31 represents the number of Chinese provinces considered in this study; *c* = 140 refers to the number of economies we include (excluding 31 Chinese provinces).

To calculate the impact of trade on hazardous waste generation in China, we extend the above nested GTAP-MRIO framework with a waste intensity vector **w** = {*w*_*k*_}, which can be calculated by *w*_*k*_ = *v*_*k*_/*x*_*k*_, where *v*_*k*_ and *x*_*k*_ represent the total amount of hazardous waste generation and total output in individual sector *k*, respectively. Since developing a hazardous waste dataset with a uniform definition of hazardous waste and global coverage is very difficult,^60^ we focus only on hazardous waste generated in China and set the waste intensities to 0 for all other countries following previous studies.^61^

The consumption- and production-based hazardous waste generation of different Chinese provinces can be estimated using eqs 5 and 6. Specifically, consumption-based hazardous waste generation (*G*_consumption_^*r*^) refers to hazardous waste driven by the final demand of province *r*; while production-based hazardous waste generation (*G*_production_^*r*^) indicates the hazardous waste in province *r* induced by the final demand of all regions.
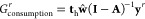
5
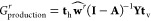
6where **t**_h_ and **t**_v_, respectively, represent the row and column vectors with all cells equal to 1; **ŵ** denotes the diagonal matrix of the waste intensity vector **w**; and  denotes the diagonal matrix of province *r*’s waste intensity vector **w**^*r*^ with corresponding hazardous waste generation intensities for all sectors in province *r* but zeroes for sectors in other regions.

Then, we quantify the trade-related hazardous waste generation in different provinces as follows:
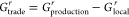
7
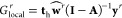
8where *G*_trade_^*r*^ measures province *r*’s trade-related hazardous waste (i.e., hazardous waste generated by local production for external final demands) and *G*_local_^*r*^ measures province *r*’s hazardous waste generated during locally consumed production (i.e., hazardous waste generated by local production for internal final demands in province *r*).

To identify critical trade flows, we further evaluate hazardous waste embodied in commodities produced in different Chinese provinces but ultimately consumed in other economies. We also quantify the virtual hazardous waste flows between different provinces in China. Equations are as follows
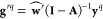
9
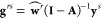
10where **g**^*rq*^ is a column vector of virtual transfer of international hazardous waste in trade, whose nonzero elements represent the sectoral hazardous waste embodied in trade from province *r* (the producer) to country/region *q* (the consumer); similarly, **g**^*rs*^ is a column vector of trade-induced hazardous waste transfer within China, whose nonzero elements reflect the sectoral hazardous waste embodied in trade from province *r* (the producer) to province *s* (the consumer).

## 3 Results and Discussion

### 3.1 Unequal Consumption- and Production-Based Hazardous Waste

China’s industrial production generated 39.76 million tons (mt) of hazardous waste in 2015, approximately 10% of the world’s total.^7^ Manufacture of chemical products (CHEMI), smelting & processing of metals (SM_ME), mining and processing of nonmetal and other ores (MN_NM), and manufacture of paper and printing (PAPER) are the main sectors that generate hazardous wastes, accounting for 71% of China’s total hazardous waste generation in that year. More details on sectoral contributions to hazardous waste can be found in Figure S1 and Table S3 in the Supporting Materials.

**Figure 1:**
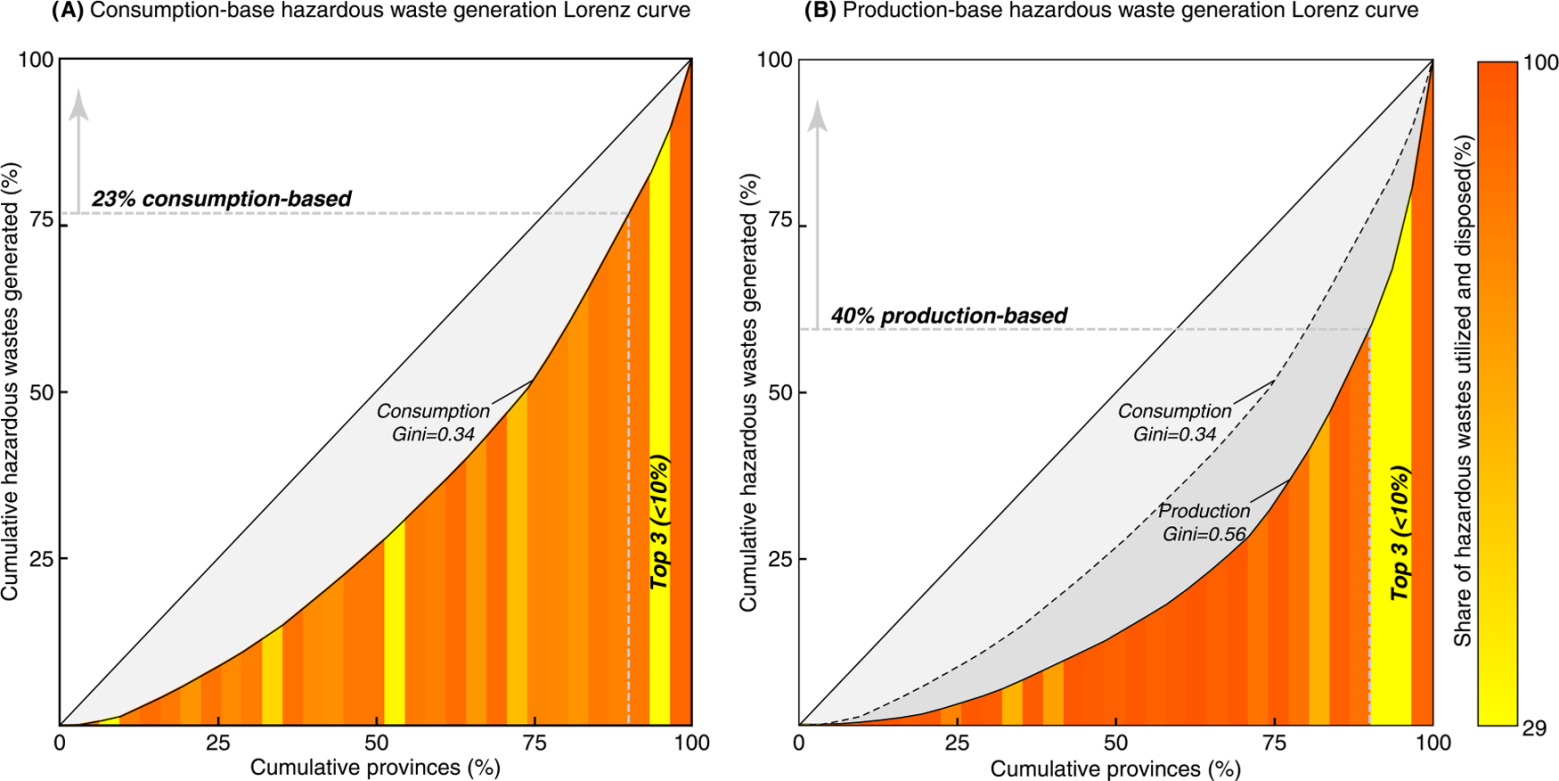
Lorenz curves for consumption-based (A) and production-based (B) hazardous waste generation of 31 Chinese provinces. For consumption- and production-based Lorenz curves, provinces are sorted by consumption- and production-based hazardous waste generation from the lowest on the left to the highest on the right, respectively.

**Figure 2:**
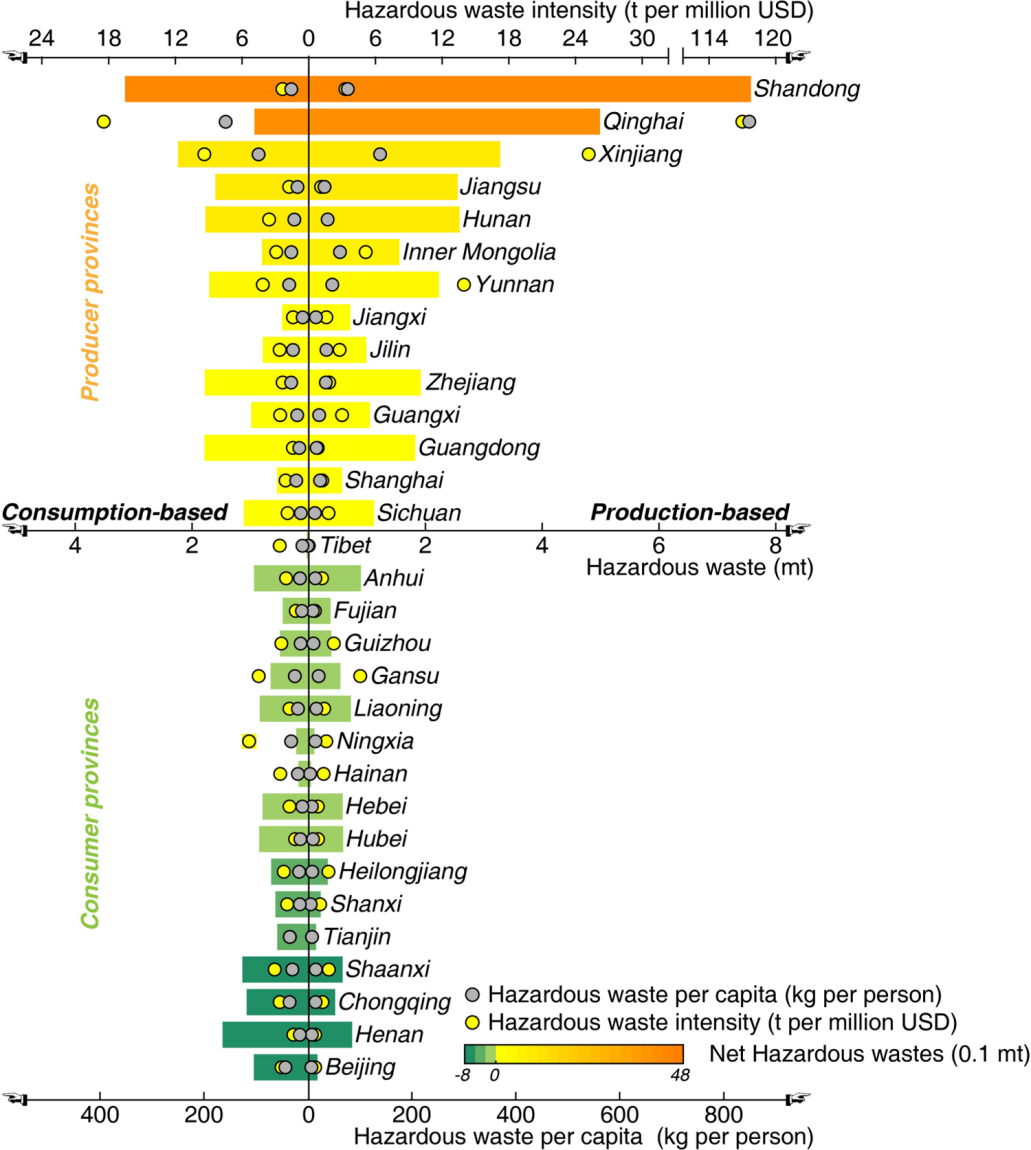
Consumption- and production-based hazardous waste generation. The length of the bars illustrates the consumption- and production-based hazardous waste generated in each province. The bars are ranked and color-coded according to the amount of net hazardous waste generation from green (consumer provinces, consumption-based waste > production-based waste) to orange (producer provinces, production-based waste > consumption-based waste).

From a consumption perspective, there is a huge disparity in hazardous waste generation across provinces (shown in Figure 1A and the length of bars on the left in Figure 2). The top 10% provinces are responsible for 23% of the national total, while the bottom half contributes only 28%. The greatest consumption-based contributors are Shandong (3.15 mt, 10% of the total), Xinjiang (2.24 mt, 7%), and Guangdong (1.79 mt, 6%).

From the production perspective, the disparity of hazardous waste among provinces is even starker. Figure 1 shows Lorenz curves for consumption- and production-based hazardous waste generation in 31 Chinese provinces. The horizontal axis represents the cumulative share of provinces, and the vertical axis shows the cumulative share of consumption- and production-based hazardous waste generation. As shown in Figure 1B, the top 10% provinces generated 40% of China’s industrial hazardous waste in 2015 (19% in Shandong, 13% in Qinghai, and 8% in Xinjiang). In contrast, provinces ranked in the bottom 50% produce only 14% of China’s hazardous waste, with each accounting for less than 2%. Furthermore, we calculate the Gini coefficient (i.e., the ratio of the area between the perfect equality line and the Lorenz curve divided by the area below the diagonal, see the Supporting Materials for details) to measure the inequalities of hazardous waste generation across provinces.^62^ The results show that the Gini coefficient from the production perspective is as high as 0.56, 1.65 times that on the consumption side. Such exacerbated inequalities in provinces’ production-based hazardous waste are also captured when looking at per capita hazardous waste generation and waste generation intensities (measured by hazardous waste generation per unit of industrial output, shown in Figure 2). Taking Qinghai, the province with the highest per capita hazardous waste generation and intensities from both perspectives as examples, consumption-based per capita hazardous waste generation and hazardous waste intensity in Qinghai are 5 and 8 times the national average, while the numbers on the production side soar to an astonishing 28 and 54, respectively.

Notably, the extreme inequality on the production side poses great challenges to waste management in hotspot provinces, especially in their key industries (e.g., manufacture of paper, printing (PAPER) in Shandong and mining and processing of nonmetal and other ores (MN_NM) in Qinghai and Xinjiang). More details on sectoral contributions to each province’s hazardous waste are shown in Figure S2 in the Supporting Materials. In recent years, hazardous wastes from all sectors in China must be reused or disposed of at specialized facilities with hazardous waste operation permits (mainly including incineration, landfilling, etc.).^6,13^ However, in general, these facilities do not target specific types of hazardous waste but are able to reduce or eliminate the hazards of a relatively wide range of wastes.^63^ The colors below the Lorenz curves in Figure 1 indicate the share of hazardous waste reused and disposed for the corresponding province from low (yellow) to high (orange). We find that in nearly 2/3 of Chinese provinces, more than 95% of the total generated hazardous waste can be reused or disposed of. However, shockingly, for two of China’s top 3 hazardous waste producers, only around 1/3 of hazardous waste generated in 2015 was reused or disposed (29% in Qinghai, 38% in Xinjiang) due to their insufficient treatment capacities. Moreover, given the current production of such waste and limited capacities for reusing or processing, stockpiles of hazardous waste will continue to rise over time, threatening the surrounding environment and the well-being of residents.^12,13^

### 3.2 Impact of Trade on Hazardous Waste in China

Figure 2 also indicates a significant mismatch between consumption- and production-based hazardous waste generation. Fourteen provinces are defined as producer provinces where production-based hazardous waste is higher than consumption-based waste. In contrast, the remaining 17 are consumer provinces that induce much more hazardous waste than they generate within their geographical boundaries. It is noteworthy that consumption-based hazardous waste generation is 4–7 times higher than the corresponding production-based generation in Beijing, Hainan, and Tianjin, which are either developed megacities or islands and are dominated by tertiary industry.^12^ These consumer provinces are often overlooked in hazardous waste management on the production side, despite their roles as vital drivers.

**Figure 3:**
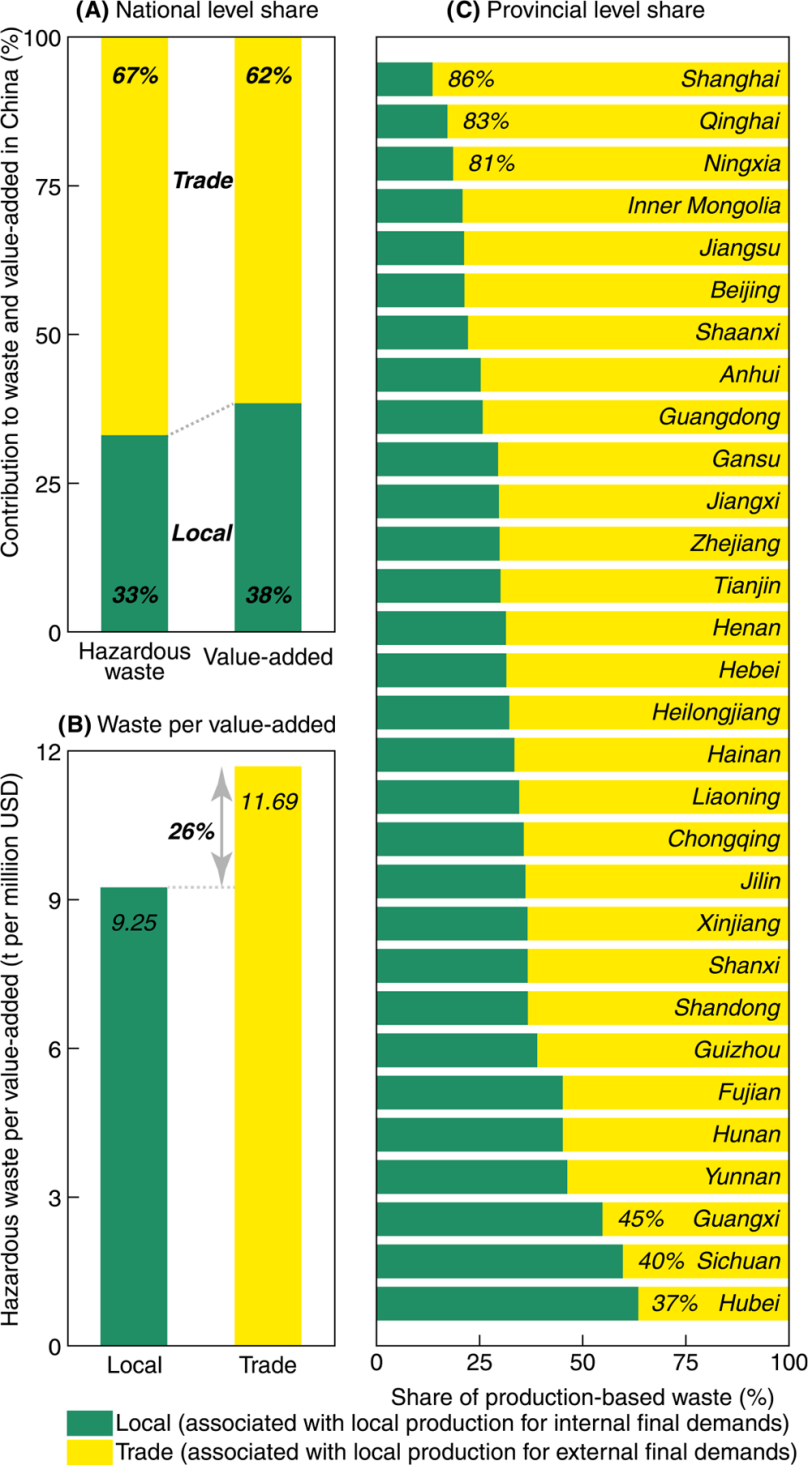
Impact of trade on China’s hazardous waste generation. (A) Impact of trade on national hazardous waste generation and value-added. (B) Hazardous waste per value-added associated with local production for internal (local) and external final demands (trade). (C) Impact of trade on provincial hazardous waste generation.

The separation of waste-intensive commodities production from their consumption is induced by expanding interregional trade.^64^Figure 3A shows a dramatic spatial shift in China’s industrial hazardous waste generation facilitated by trade. In 2015, 67% of the industrial hazardous waste in China was generated during the production of goods that were ultimately consumed in another province or even abroad. The proportion is significantly higher than the results of other environmental consequences found by previous studies (e.g., 50% for carbon emissions in 2012,^65^ 50–60% for air pollutant emissions in 2007^66^) and is 5% points higher than the share of industrial value-added embodied in trade. For more than 90% of provinces, trade-related (i.e., local production for external final demands) hazardous waste generation accounts for over half of the local generation (Figure 3C). Shanghai, Qinghai, and Ningxia are the three provinces most severely affected by export production, with a proportion of trade-related hazardous waste generation exceeding 80% (86% for Shanghai, 83% for Qinghai, and 81% for Ningxia). Even in the least-affected province (i.e., Hubei), the contribution of trade-related production is still as high as 37%.

In addition, trade-related production is more waste-intensive than locally consumed production (i.e., local production for internal final demand, shown in Figure 3B). Industrial activities serving local consumption produce 9.25 t of hazardous waste per one million USD value-added, whereas trade-related production generates an average of 11.69 t (i.e., 26% more) of hazardous waste to achieve the same economic outcome. For around 2/3 of Chinese provinces, producing for external final demands is generally more waste-intensive than for their own final demands. Notably, in Qinghai, driven by mining and processing of nonmetal and other ores (MN_NM), trade-related production generates 145% higher hazardous waste per value-added than locally consumed production. More details of the hazardous waste per value-added for each province can be found in Figure S3. Hence, at the national and provincial levels, in terms of both volume and intensity, our results highlight the necessity for an in-depth understanding of trade-related hazardous waste generation.

### 3.3 Hazardous Waste Driven by Overseas Consumers

Although the Basel Convention and the Chinese import ban prohibit transboundary movements of hazardous waste to China for any purpose, other countries still de facto transfer enormous (but virtual) hazardous waste to China. In 2015, overseas final demand resulted in 8.30 mt of hazardous waste in China, accounting for 21% of China’s total industrial hazardous waste generation that year.

**Figure 4:**
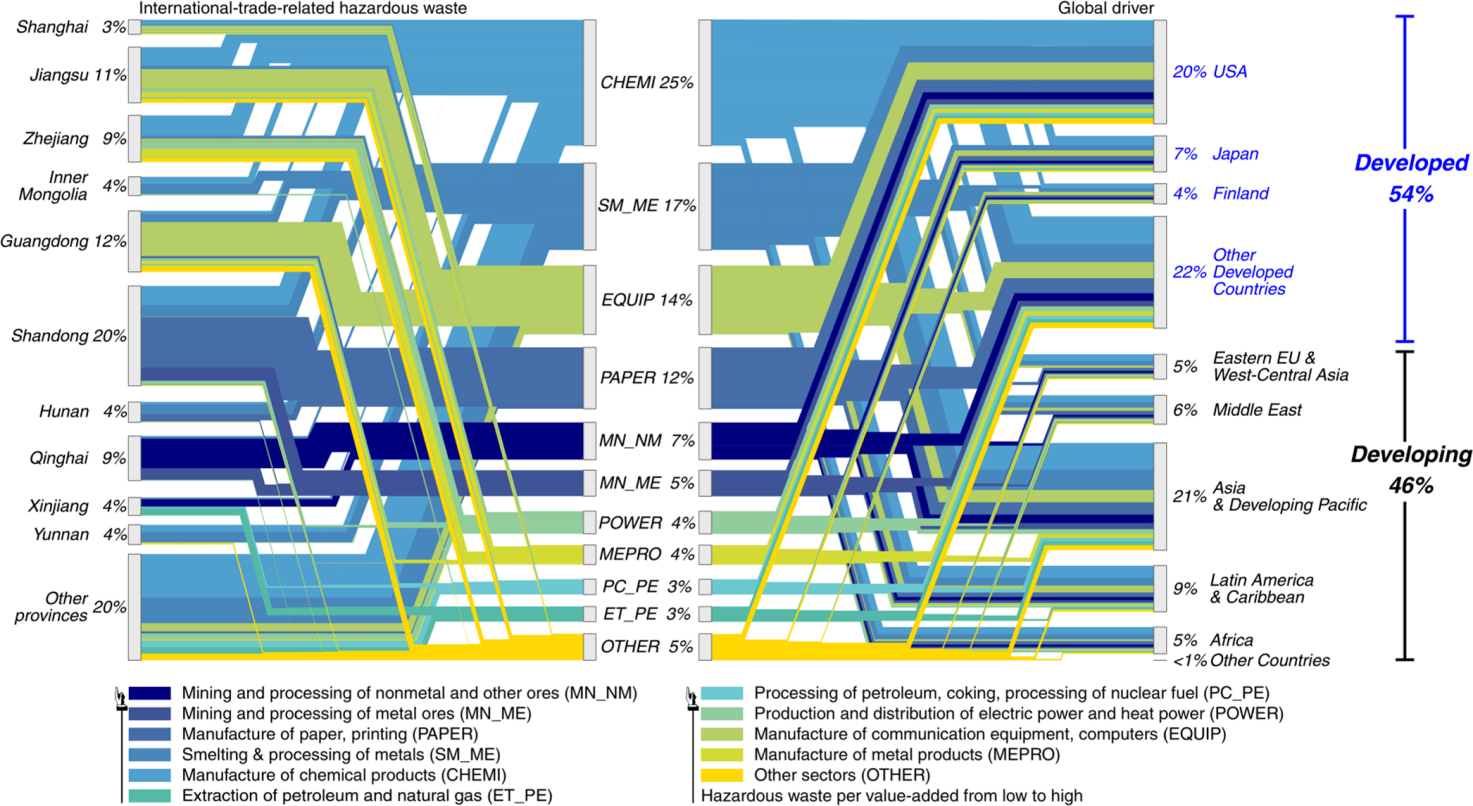
International-trade-related virtual hazardous waste flows. The provinces on the left and the sectors in the middle show the top 10 provinces and sectors with the largest international-trade-related hazardous waste generation and an aggregation of the rest. Overseas consumers on the right include the top 3 countries that induced the largest hazardous waste in China, and the rest are grouped according to the United Nation’s country classification.

Figure 4 details the flow of hazardous waste relating to China’s exports. 54% of international-trade-related hazardous waste generated in China is attributed to developed countries. The United States is the largest overseas driver, and its final consumption alone triggers 1.70 mt (or 4%) of hazardous waste in China, followed by Japan and Finland, inducing 7% and 4% of the nation’s international-trade-related hazardous waste, respectively. 70% of China’s international-trade-related hazardous waste is concentrated in the manufacture of chemical products (CHEMI), smelting and processing metals (SM_ME), manufacture of communication equipment, computers (EQUIP), and manufacture of paper, printing (PAPER).

Provinces with the largest international-trade-related hazardous waste include China’s inland provinces with relatively low GDP per capita (such as Yunnan, Xinjiang, Qinghai, and Ningxia), as well as the richest coastal provinces (such as Shanghai, Jiangsu, Zhejiang, and Guangdong). Nevertheless, such virtual dumping through international trade is unfair to less-developed provinces, since they obtain fewer economic benefits at the same cost of hazardous waste generation. Provinces in Figure 4 are ranked by GDP per capita from low (bottom) to high (top). The streams are color-coded to reflect international-trade-related hazardous waste generated per unit of value-added in each sector, from yellow (low) to blue (high). Specifically, high-income provinces primarily supply high-value-added products generating relatively low hazardous waste per unit of value-added to overseas consumers. For instance, in Jiangsu and Zhejiang, manufacture of communication equipment, computers (EQUIP) produces the largest share of hazardous waste for overseas consumers (36% for Jiangsu, and 56% for Zhejiang). In contrast, in less-developed provinces, relatively waste-intensive industries are the major contributors to waste induced by overseas consumption. For instance, 67% of the international-trade-related hazardous waste in Qinghai is generated in mining and processing of nonmetal and other ores (MN_NM). The value-added per unit output in mining and processing of nonmetal and other ores (MN_NM) sector is, on average, less than 1/17 that of the leading sector in Jiangsu and Zhejiang (manufacture of communication equipment, computers (EQUIP) sector).

Revealing virtual hazardous waste transfers resulting from international trade may be as necessary as controlling physical transboundary movements between nations. On the one hand, the scale of international trade-embodied transfers is larger than that of physical ones. For example, according to the Basel Convention’s national reporting dataset, Finland directly exports 122,303 t of waste with hazardous properties to other countries in 2015.^67^ In comparison, the leakage of hazardous waste from Finland via global trade to a single country, China, is almost 3 times the total physical outflows from Finland. And the gap is likely to widen, as trade expands and regulations on the physical movements of hazardous waste become more stringent.^7,64^ On the other hand, trade-driven relocation of hazardous waste requires a broader concept of environmental responsibility. Although physical transboundary movements successfully shift the potential risk associated with disposal elsewhere, the exporters still need to bear the relevant economic costs and ensure that the importer does manage the wastes in compliance with local laws and regulations, whereas the disposal duty of trade-embodied waste can be entirely passed on to other regions (i.e., the producers), as the responsibility for proper treatment of hazardous waste belongs to the entities generating them.^34,67^ Thus, expanding the scope of international regulations, such as the Basel Convention, by enhancing consumer responsibilities under a virtual hazardous waste network, is of great importance.

### 3.4 Displacement of Hazardous Waste through Interprovincial Trade in China

Hazardous waste embodied in China’s domestic trade between 31 provinces is greater than those caused by overseas consumers. Specifically, 18.32 mt of hazardous waste is associated with the production of goods that are ultimately consumed in another province, exceeding 2 times the amount of hazardous waste driven by overseas consumers. Here, hazardous waste per unit of value-added is 36% higher than hazardous waste related to international trade.

**Figure 5:**
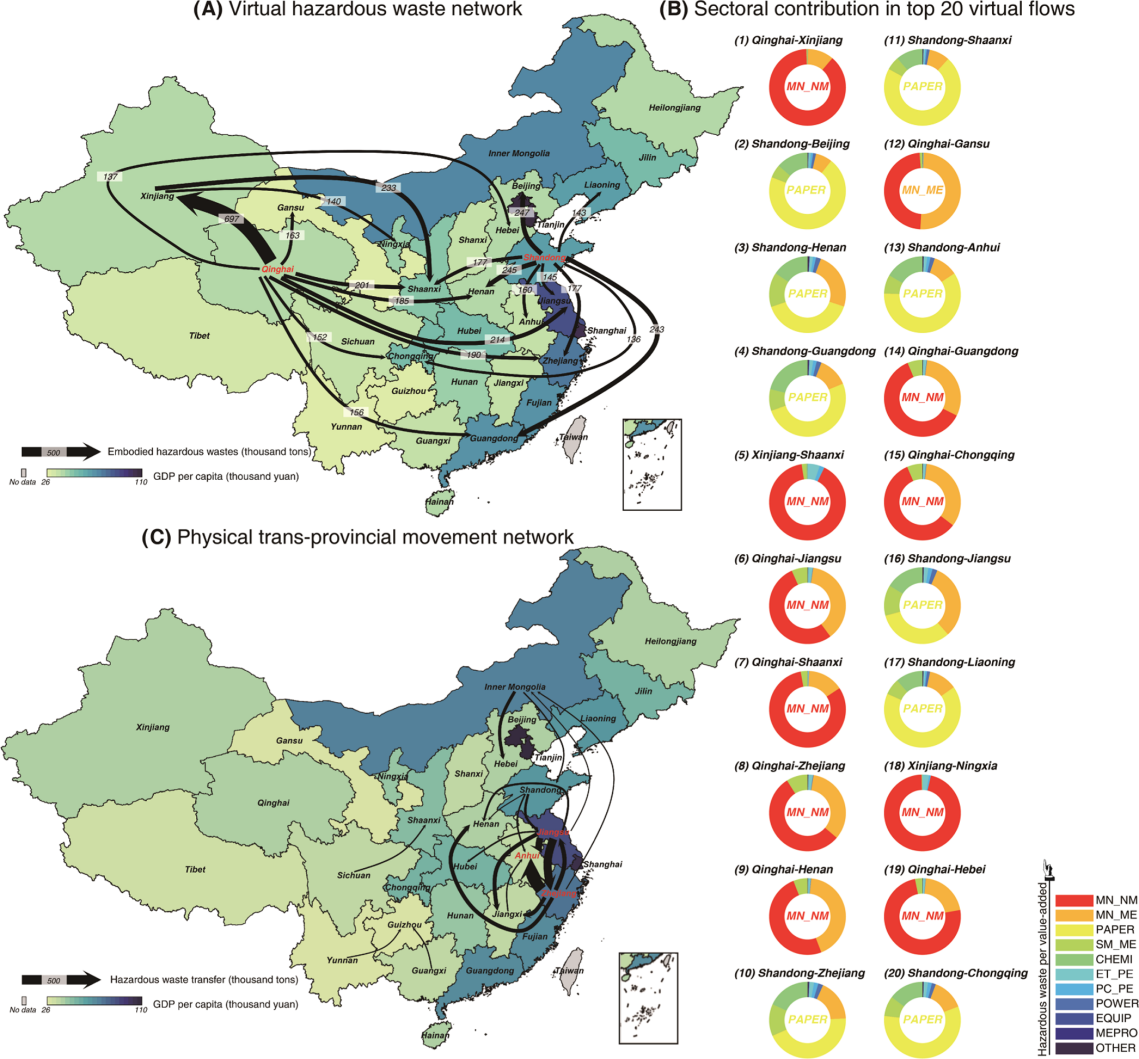
Interprovincial hazardous waste network in China. (A) Virtual hazardous waste network in China’s domestic trade. (B) Sectoral contribution in the top 20 virtual flows. (C) Physical trans-provincial movements network. The top 20 pairs for hazardous waste outsourcing and physical trans-provincial movements are presented.

Figure 5A presents the virtual hazardous waste network caused by interprovincial trade in China. Compared with physical trans-provincial movements of hazardous waste (shown in Figure 5C), virtual waste transfers triggered by China’s interprovincial trade show several unique characteristics. First, the scale of virtual transfers across provinces in China is larger than that of physical ones. Virtual transfers of hazardous waste in China’s domestic trade are an order of magnitude higher than those directly caused by trans-provincial movements. Second, physical movements of hazardous waste occur mostly between the more developed provinces in the Yangtze River Delta. They are mainly short-distance shipments between neighboring provinces, while the major virtual transfers span greater distances and involve more provinces. Among the top 20 virtual hazardous waste outsourcing pairs, 9 outflows are from Shandong, and 9 are from Qinghai. The largest origin-destination pair supporting the virtual network is Qinghai-Xinjiang, where the amount of embodied hazardous waste is higher than the sum of the generation in Hainan, Ningxia, Tianjin, Beijing, and Shaanxi that year. Hazardous waste embodied in trade from Shandong to Beijing, Henan, and Guangdong is around 240,000 t, ranking at the forefront as well. Thirdly, from initial generation to eventual disposal, the whole process of physical trans-provincial movements is strictly monitored by the ecology and environment departments.^68^ In contrast, the virtual waste transfers triggered by China’s interprovincial trade have received little attention from policymakers. All of these characteristics determine that managing the virtual hazardous waste network caused by domestic trade in China is urgent and more complicated.

Hazardous waste outflows occur mainly when a province produces something that is used throughout the country and generates hazardous waste. The main contributing sectors of the virtual interprovincial flows of hazardous waste are highly consistent with the types of hazardous waste generating industry dominating different provinces. Figure 5B provides the sectoral contribution of the top 20 virtual hazardous waste flows. Hazardous waste has the highest proportion (32–71%) in all top virtual flows leaving Shandong. The multiple tailings generated by mining and processing of nonmetal and other ores (MN_NM) account for more than half of most of the top virtual flows from Qinghai and Xinjiang (48–96%). Therefore, uncovering the virtual transfer network of hazardous waste generation between provinces may provide essential insights into waste management for critical provinces from the production side.

Promoting the transfer of advanced production technologies^69^ suggests a potential path toward hazardous waste minimization in the key industries in hotspots. For example, among the domestic-trade-related hazardous waste in Shandong’s manufacture of paper, printing (PAPER), Beijing, Shaanxi, and Guangdong contribute 174 thousand t (10%), 126 thousand t (7%), and 124 thousand t (6%), respectively. It is worth noting that the hazardous waste intensity in manufacture of paper, printing (PAPER) sector in Shandong is significantly higher than in other provinces. If Shandong’s production-based hazardous waste intensity in that sector was at the level of the consumer provinces (see the Supporting Information for details), then Shandong’s production-based hazardous waste generation could be reduced by 22% (1.68 mt) from the current level.

However, for some areas primarily relying on their unique mineral resource endowments and relevant production, the applicability of the above strategies is likely limited. In such cases, consumer responsibilities should be emphasized during the treatment process. Here, we take mining and processing of nonmetal and other ores (MN_NM) sector in Qinghai as an example. The neighboring Xinjiang is the largest driver of these wastes, with a proportion of 27%. Other major consumers include Shaanxi, Jiangsu, Zhejiang, Hebei, Guangdong, and Henan, each contributing 4–8%. Embodied hazardous waste outflowing from Qinghai’s mining and processing of nonmetal and other ores (MN_NM) sector are mainly asbestos mine tailings,^70^ which have been identified as carcinogens by the International Agency for Research on Cancer (IARC).^71^ Due to the lack of treatment capacity, only 1/3 of the hazardous waste generated in Qinghai’s mining and processing of nonmetal and other ores (MN_NM) sector is reused or disposed. In addition, asbestos tailings have been accumulating over the years. As a result, the total amount of asbestos waste stored in 2015 is 16 times its annual generation. 42% of China’s known reserves of asbestos minerals are located in Qinghai.^72^ Lacking production experience in asbestos mining, technical assistance to Qinghai from other provinces may be very limited. To address the waste issues in such an area, consuming provinces, especially the distant eastern provinces, may improve Qinghai’s overall hazardous waste disposal capacity by investing in general hazardous waste disposal projects (e.g., incinerators, landfills), or promoting integrated reuse projects for a specific type of hazardous waste (e.g., extracting magnets from asbestos tailings) in Qinghai. Meanwhile, to improve the efficiency of national hazardous waste disposal, the Chinese government has recently been encouraging interprovincial sharing of hazardous waste centralized disposal capabilities.^73^ In this context, neighboring provinces may also share Qinghai’s disposal pressure by allowing Qinghai to transport hazardous waste to their vacant facilities for disposal.

To increase the willingness of other provinces to implement consumer responsibilities via the above-mentioned routes, the central government may build an operating mode similar to the well-known “Clean Development Mechanism (CDM)” in reducing carbon emissions. That is to say, efforts by investing in general disposal projects and targeted hazardous waste reuse projects in other provinces, or by transporting hazardous waste from other provinces to assist their disposal can be counted toward meeting the hazardous waste reduction targets of each province.^74^ Notably, differences in the environmental benefits of reducing different types of hazardous waste should be considered in policy innovation.

### 3.5 Limitations

This study is subject to some limitations and uncertainties. First, incomplete hazardous waste data may introduce uncertainties to trade-embodied hazardous waste estimation. The CESD incorporates self-reported waste information from all registered industrial enterprises generating hazardous waste. This data collection scheme implies possible sample omission issues, ultimately ending up with an underestimation of hazardous waste generation. Meanwhile, companies may intentionally conceal or underreport the amount of hazardous waste they generate to reduce associated treatment costs. In response, the Chinese government has taken measures, such as enforcing the ban on informal operations via Central Environmental Inspections and strengthening data quality management at multiple stages of data collection. Currently, the CESD is regarded as the best available and the most reliable data reflecting hazardous waste generation in China.^75−77^ With the development of China’s whole life cycle management of hazardous waste, such uncertainties will be gradually reduced.

Another limitation of this study comes from the globally integrated MRIO table. Many studies assume that China’s imports from different countries hold the same production structure and technology as China’s domestic structure. To avoid the considerable uncertainties from this assumption, we employed the globally integrated MRIO table developed by Zheng et al.^41^ It combines two widely used MRIO tables (i.e., China’s 2015 MRIO table from Carbon Emission Accounts & Datasets (CEADs)^78^ and the GTAP-MRIO table^79,80^) to enable differentiated production structures, technologies, and hazardous waste intensities in different countries, thereby reducing the associated uncertainties. When more detailed statistics become available, the uncertainties from the globally integrated MRIO model may be further reduced.

Our study focuses on hazardous waste in China, analyzing hazardous waste embodied in trade. Despite the sheer volume, the results of this study are only one piece of the global virtual hazardous waste transfer puzzle. We do acknowledge that it would be preferable to characterize virtual transfer networks worldwide. However, there is only a descriptive definition of hazardous waste globally (i.e., waste that is toxic, corrosive, ignitable, reactive, or infectious, causing harmful effects on human health and the environment).^67^ As a result, different economies scope hazardous wastes through their own identification processes, resulting in different catalogues of hazardous wastes.^6,81^ Furthermore, hazardous waste information is usually reported in highly aggregated categories; thus, integrating datasets developed by different countries using a uniform definition is very difficult.^60^ Simply linking hazardous waste statistics in different countries to enable a global study may lead to significant bias. Therefore, in this proof-of-concept study, this research assesses the impact of international and interprovincial trades on China’s hazardous waste generation. Given that China is the world’s largest waste producer,^12,13^ our Chinese case can provide important insights for reliable research with global coverage in the future.

Following previous studies,^61,82^ we assume that the waste intensities of other economies are zero in consumption-based accounting. This may lead to an underestimation of China’s consumption-based hazardous wastes. Given the unavailability of global hazardous waste information, more complicated assumptions about hazardous wastes in other countries may dilute the findings based on reliable Chinese data, thereby contributing to further uncertainties. Similar assumptions have been accepted in previous studies on virtual networks of multiple environmental consequences, such as carbon emissions^61,82^ and air pollution^38,48,83^ in China.

This study distinguishes different types of hazardous waste by providing sectoral hazardous waste details. This assumes that hazardous wastes from the same sector have similar characteristics. In policy practice, the assumption in this study is widely accepted. For example, in China and the US, hazardous wastes are mainly classified according to the sectors generating them.^6,81^ However, we do acknowledge that, within a sector, the properties of hazardous waste may vary. Product-specific life cycle assessment (LCA) and hazardous-waste-specific toxicology experiments may be the perfect solution to this limitation.^84−86^ Yet a case-by-case analysis of all types of hazardous wastes across all industries is costly. With the increment of the number of available statistical variables and the development of various environmental censuses, future studies on virtual waste networks are expected to consider more detailed differences in hazardous waste categories.

## 4 Implications

Beyond the physical transboundary movement regulations, massive “dumping” of hazardous waste occurs virtually through interregional trade. In 2015, the virtual flows of hazardous waste in China’s trade were as high as 26.63 mt (67% of industrial hazardous waste generated in China). Production for trade is more waste-intensive than locally consumed production. Achieving the same economic output, trade-related production generates an average of 11.69 t of hazardous waste per one million USD value-added (i.e., 26% more than industrial activities serving local consumption).

Globally, overseas final demand leads to 8.30 mt (21% of the national total) of hazardous waste generation in China. Within China, 18.32 mt (46% of the national total) of hazardous wastes are embodied in its interprovincial trade. Under the impact of virtual flows, total hazardous waste generation and the following management duty are more unequally concentrated in some less-developed regions in China, compared with the provincial distribution on the consumption side. However, the less-developed top hazardous waste producers, Qinghai and Xinjiang, could dispose of only 29 and 38% of the hazardous waste they produced (mainly from mining and processing of nonmetal and other ores (MN_NM) sector) in 2015 without sufficient treatment facilities. As reported by the Chinese government, the cumulative stockpile of hazardous waste in the two provinces is as high as 76.07 and 23.99 mt in 2020, increasingly threatening the environment and impacting public health.

There is an urgent need to expand the scope of transboundary waste regulations, given that virtual transfers of hazardous waste are on a larger scale, span greater distances, and transfer waste management responsibilities more radically than physical ones. In other words, these regulations should further focus on the virtual flows of hazardous waste between economies, in addition to existing restrictions on physical transfers. Our results provide a quantitative basis for implementing consumer responsibility in various transboundary waste regulations. First, new amendments can facilitate the transfer of advanced production technologies based on virtual flows of hazardous waste, which may help key industries in critical areas to minimize hazardous waste. Second, addressing the waste challenges in hotspot producer regions that rely primarily on their unique resource endowments and relevant production, newly revised regulations can encourage corresponding consumers to fulfill their consumer responsibilities by investing in comprehensive reuse projects, building landfills, and sharing disposal capacities. Meanwhile, global authorities (e.g., the United Nations Environment Programme, UNEP) and national central governments could initiate mechanisms similar to CDM in carbon emission reduction (i.e., acknowledging the progress made in reducing and disposing of hazardous waste in other regions). Third, revised regulations can also encourage the implementation of economic measures, such as waste tariffs or environmental taxes, to stimulate changes in consumption and production patterns^87^ and thereby address hazardous waste issues at the source.

## Supporting Information Available

Figures dealing with national hazardous waste generation by sector, provincial production- and consumption-based hazardous waste generation by sector, hazardous waste per value-added associated with local production for each province’s internal and external final demands, hazardous waste generation and economic development by province in 2015, detailed information about Gini coefficient for consumption- and production-based hazardous waste generation of 31 Chinese provinces, unexpected net producers of hazardous waste, and scenario analysis on the technological improvements in Shandong’s PAPER sector (PDF)
Classification of Chinese provinces and corresponding sectors in the MRIO table used in this study, bridge between the 4-digit National Standard Industrial Classification (NSIC) industries and the aggregated MRIO sectors, hazardous waste inventory 2015, classification of other economies and corresponding economic sectors in the MRIO table used in this study, the original results including consumption- and production-based hazardous waste by province, impacts of trade on hazardous waste generation, hazardous waste per value-added associated with local production for each province’s internal and external final demands, international virtual hazardous waste network, and inter-provincial virtual hazardous waste network in China (XLSX)
