# Supplementary material for: Large Virtual Transboundary Hazardous Waste Flows: The Case of China
Source: Environ Sci Technol. 2023 May 16;57(21):8161–73. doi: 10.1021/acs.est.2c07962 (PMC10233792; doi:10.1021/acs.est.2c07962)
Supplement: Supplementary file 1 — es2c07962_si_001.pdf [file es2c07962_si_001.pdf]

Supplementary Materials for

Large virtual transboundary hazardous waste flows: the case of China

Ruoqi Li,[1] Miaomiao Liu,[1,] Yuli Shan,[2,*] Yufan Shi,[1] Heran Zheng,[3] Wei Zhang,[4] Jianxun Yang,[1] Wen Fang,[1] Zongwei Ma,[1] Jinnan Wang,[1,4] Jun Bi,[1] Klaus Hubacek,[5]*

1. State Key Laboratory of Pollution Control and Resource Reuse, School of the Environment, Nanjing University, Nanjing 210023, People's Republic of China
2. School of Geography, Earth and Environmental Sciences, University of Birmingham, Birmingham B15 2TT, UK
3. The Bartlett School of Sustainable Construction, University College London, London WC1E 7HB, UK
4. State Environmental Protection Key Laboratory of Environmental Planning and Policy Simulation, Chinese Academy of Environmental Planning, Beijing 100041, People's Republic of China
5. Integrated Research on Energy, Environment and Society (IREES), Energy and Sustainability Research Institute Groningen (ESRIG), University of Groningen, Groningen 9747 AG, the Netherlands

*** Corresponding Authors**

liumm@nju.edu.cn (M. L.); y.shan@bham.ac.uk (Y. S.)

Supplementary texts S3

Text S1. Gini coefficient for consumption- and production-based hazardous waste generation of 31 Chinese provinces. S3

Text S2. Unexpected net producers of hazardous waste. S4

Text S3. Scenario analysis on the technological improvements in Shandong's PAPER sector. S5

Supplementary Figures S6

Figure S1. National hazardous waste generation by sector. S6

Figure S2. Provincial production- and consumption-based hazardous waste generation by sector. S7

Figure S3. Hazardous waste per value-added associated with local production for each province's internal (local) and external final demands (trade). S8

Figure S4. Hazardous waste generation and economic development by province in 2015. .... S9

Supplementary Tables S10

Table S1. Classification of Chinese provinces and corresponding sectors in the MRIO table used in this study. S10

Table S2. Bridge between the 4-digit National Standard Industrial Classification (NSIC) industries and the aggregated MRIO sectors. S10

Table S3. Hazardous waste inventory 2015. .... S10

Table S4. Classification of other economies and corresponding economic sectors in the MRIO table used in this study. S10

Table S5. Consumption- and production-based hazardous waste by province. S10

Table S6. Impacts of trade on hazardous waste generation. S10

Table S7. Hazardous waste per value-added associated with local production for each province's internal and external final demands. S10

Table S8. International virtual hazardous waste network. S10

Table S9. Inter-provincial virtual hazardous waste network in China. S10

Supplementary texts

Text S1. Gini coefficient for consumption- and production-based hazardous waste generation of 31 Chinese provinces.

Gini proposes the Gini coefficient to quantify the inequalities in income. It is usually calculated based on the corresponding Lorenz curve [1]. In this study, the Gini coefficients for consumption- and production-based hazardous waste generation of 31 Chinese provinces are calculated as follows:

$$G = 1 - \left| \sum_{r=1}^{p} (CP_{r+1} - CP_r)(CW_{r+1} + CW_r) \right|$$

where G is the Gini coefficient; CP represents the cumulative share of provinces; CW represents the cumulative share of hazardous waste generation; $p = 31$ represents the number of Chinese provinces considered in this study.

Text S2. Unexpected net producers of hazardous waste.

We notice from Figure S2-A and B that the prosperous provinces on the southeastern coast of China (namely, Shanghai, Jiangsu, Guangdong, and Zhejiang), net consumers of other environmental outcomes in previous research [2-4], are unexpectedly identified as net producers of hazardous waste in this study. This is mainly caused by the share of waste-intensive sectors, such as electronic and telecommunications equipment manufacturing, broadly regarded as environmentally friendly due to relatively low energy consumption and consequent emissions [5, 6].

Text S3. Scenario analysis on the technological improvements in Shandong's PAPER sector.

Given that the hazardous waste intensity in the PAPER sector in Shandong is significantly higher than in other provinces, we developed a scenario to analyze the potential impacts of technological improvements in Shandong's PAPER sector on hazardous waste generation. Specifically, we assume that consumer provinces will implement their consumer responsibilities by transferring their advanced technologies in the PAPER sector (i.e., replacing the hazardous waste intensity of the PAPER sector in Shandong with those of the corresponding consumer provinces, in the calculation of Shandong's local hazardous waste generation).

Supplementary Figures

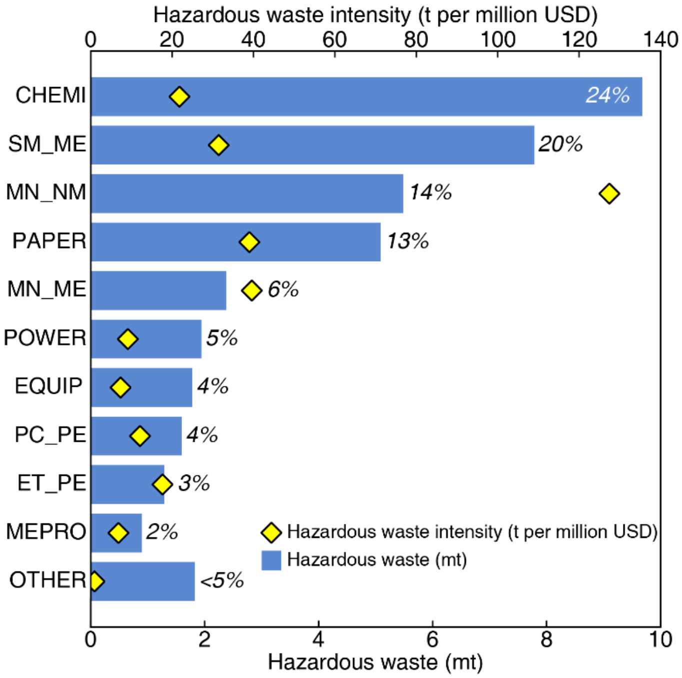

Figure S1. National hazardous waste generation by sector. The top 10 sectors generating hazardous wastes include manufacture of chemical products (CHEMI), smelting & processing of metals (SM_ME), mining and processing of nonmetal and other ores (MN_NM), manufacture of paper, printing (PAPER), mining and processing of metal ores (MN_ME), production and distribution of electric power and heat power (POWER), manufacture of communication equipment, computers (EQUIP), processing of petroleum, coking, processing of nuclear fuel (PC_PE), extraction of petroleum and natural gas (ET_PE), and manufacture of metal products (MEPRO).

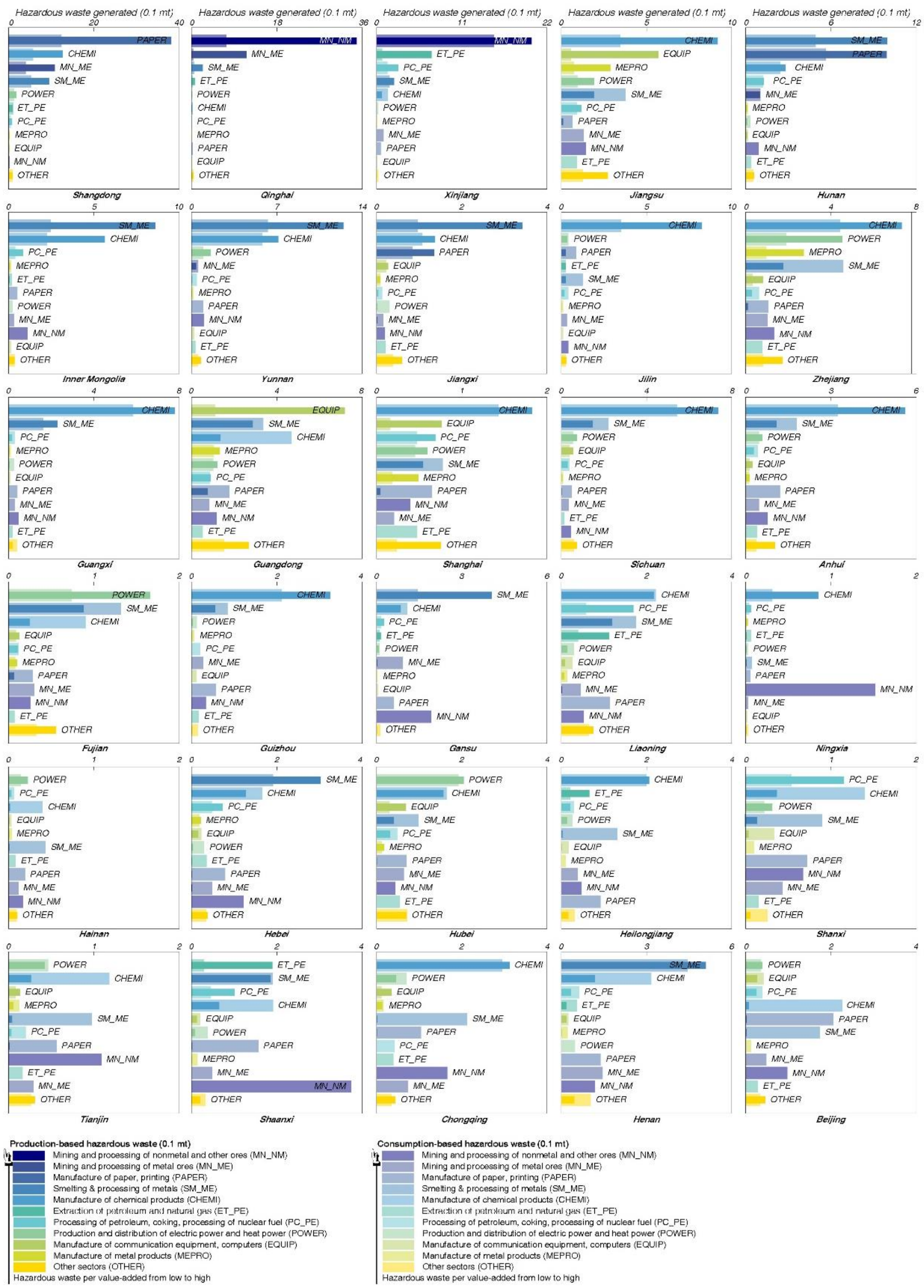

Figure S2. Provincial production- and consumption-based hazardous waste generation by sector. The provinces are ranked according to the amount of net hazardous waste generation. The top 10 sectors in the sub-figure of each province are ranked according to the amount of production-based hazardous waste.

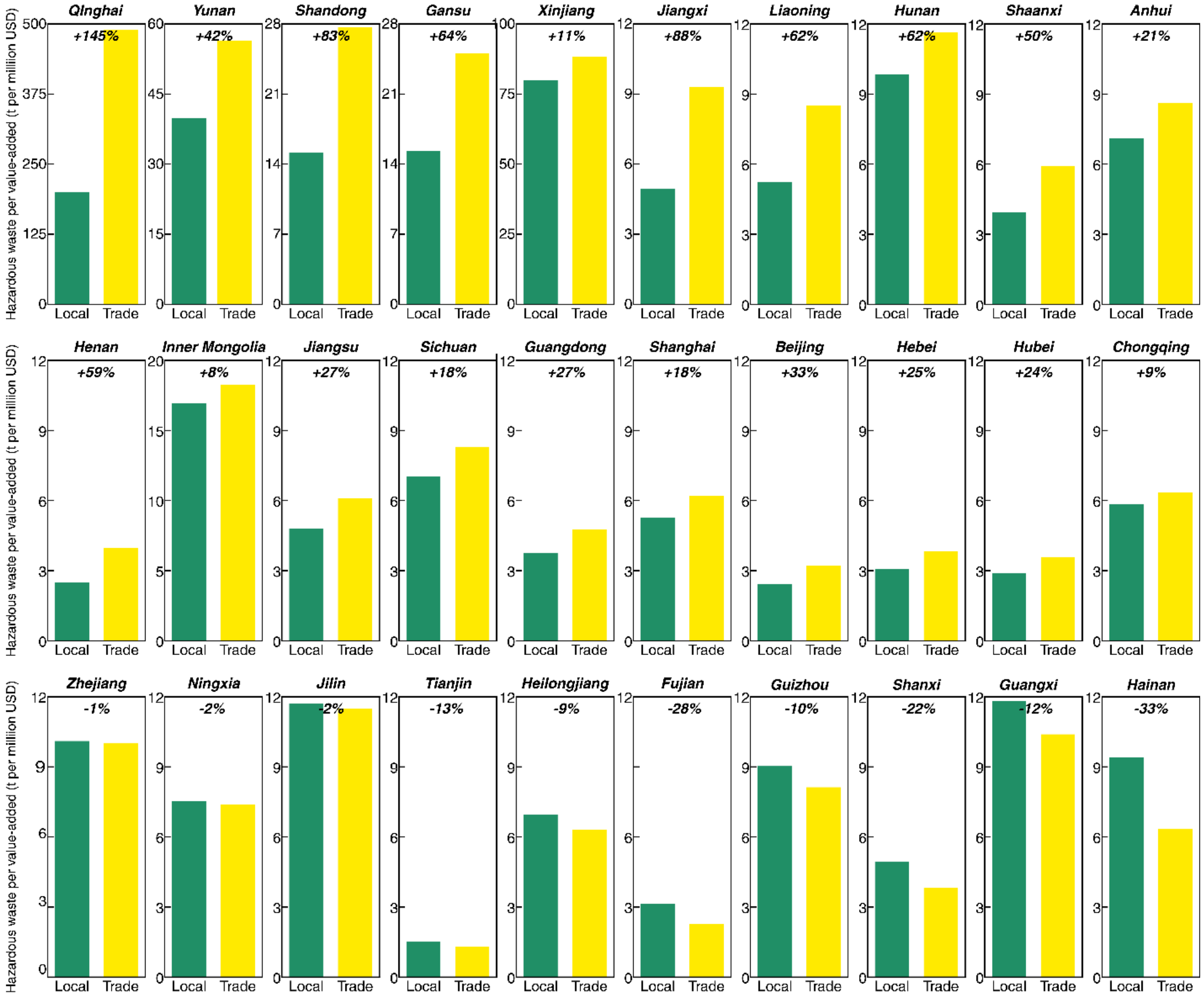

Figure S3. Hazardous waste per value-added associated with local production for each province's internal (local) and external final demands (trade). The provinces are ranked by the gap between hazardous waste per value-added associated with local production for internal and external final demands. Detailed data are shown in Table S7 in the supplementary materials.

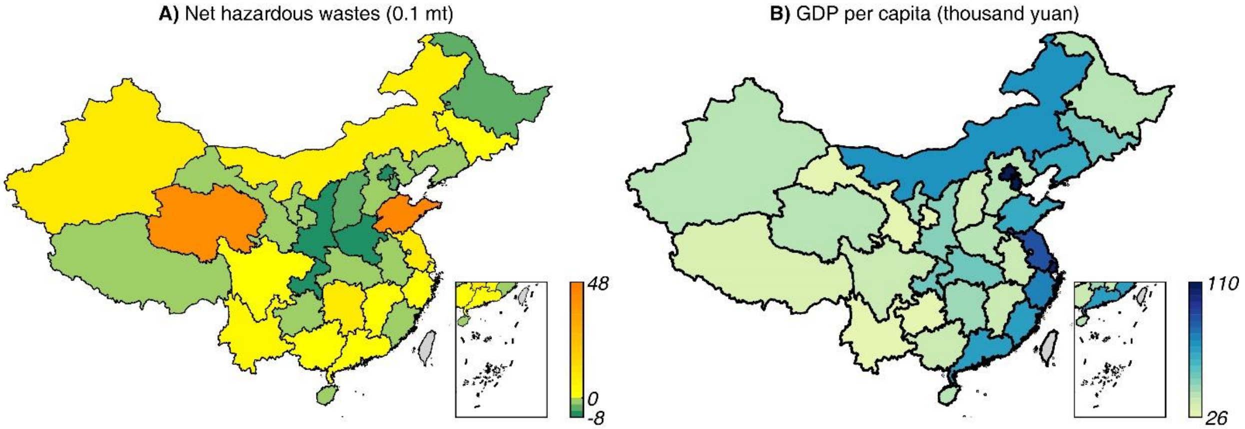

Figure S4. Hazardous waste generation and economic development by province in 2015. A) Net hazardous waste generation. B) GDP per capita.

Supplementary Tables

Table S1. Classification of Chinese provinces and corresponding sectors in the MRIO table used in this study. (see excel)

Table S2. Bridge between the 4-digit National Standard Industrial Classification (NSIC) industries and the aggregated MRIO sectors. (see excel)

Table S3. Hazardous waste inventory 2015. (see excel)

Table S4. Classification of other economies and corresponding economic sectors in the MRIO table used in this study. (see excel)

Table S5. Consumption- and production-based hazardous waste by province. (see excel)

Table S6. Impacts of trade on hazardous waste generation. (see excel)

Table S7. Hazardous waste per value-added associated with local production for each province's internal and external final demands. (see excel)

Table S8. International virtual hazardous waste network. (see excel)

Table S9. Inter-provincial virtual hazardous waste network in China. (see excel)

References

1. Shan, Y.; Guan, D.; Hubacek, K.; Zheng, B.; Davis, S. J.; Jia, L.; Liu, J.; Liu, Z.; Fromer, N.; Mi, Z.; Meng, J.; Deng, X.; Li, Y.; Lin, J.; Schroeder, H.; Weisz, H.; Schellnhuber, H. J. City-level climate change mitigation in China. *Sci. Adv.* **2018**, *4* (6), eaaq0390.
2. Mi, Z. F.; Meng, J.; Guan, D. B.; Shan, Y. L.; Song, M. L.; Wei, Y. M.; Liu, Z.; Hubacek, K. Chinese CO2 emission flows have reversed since the global financial crisis. *Nat. Commun.* **2017**, *8*, 10.
3. Zhang, W.; Liu, Y.; Feng, K.; Hubacek, K.; Wang, J.; Liu, M.; Jiang, L.; Jiang, H.; Liu, N.; Zhang, P.; Zhou, Y.; Bi, J. Revealing Environmental Inequality Hidden in China's Inter-regional Trade. *Environ. Sci. Technol.* **2018**, *52* (13), 7171-7181.
4. Zheng, H.; Zhang, Z.; Wei, W.; Song, M.; Dietzenbacher, E.; Wang, X.; Meng, J.; Shan, Y.; Ou, J.; Guan, D. Regional determinants of China's consumption-based emissions in the economic transition. *Environ. Res. Lett.* **2020**, *15* (7), 074001.
5. Guan, Y.; Shan, Y.; Huang, Q.; Chen, H.; Wang, D.; Hubacek, K. Assessment to China's Recent Emission Pattern Shifts. *Earths Future* **2021**, *9* (11), e2021EF002241.
6. Shan, Y.; Huang, Q.; Guan, D.; Hubacek, K. China CO2 emission accounts 2016-2017. *Sci. Data* **2020**, *7* (1), 54.
